# Non‐Fungi Diversity Present in Soil and Snow Impacted by Anthropogenic Activity in King George Island, Maritime Antarctica Using Metabarcoding Approach

**DOI:** 10.1111/jeu.70122

**Published:** 2026-09-11

**Authors:** Fábio Leal Viana Bones, Carlos Henrique Russi, Fabyano Alvares Cardoso Lopes, Micheline Carvalho‐Silva, Peter Convey, Luiz Henrique Rosa, Paulo Eduardo Aguiar Saraiva Câmara

**Affiliations:** ^1^ Pós‐Graduação em Fungos, Algas e Plantas Universidade Federal de Santa Catarina Florianópolis SC Brazil; ^2^ Instituto Nacional de Pesquisa Amazônica (INPA) Pós‐Graduação em Ecologia Manaus Brazil; ^3^ Laboratório de Microbiologia Universidade Federal do Tocantins Porto Nacional TO Brazil; ^4^ Departamento de Botânica Universidade de Brasília Brasília DF Brazil; ^5^ British Antarctic Survey Natural Environment Research Council Cambridge UK; ^6^ Department of Zoology University of Johannesburg Auckland Park South Africa; ^7^ Millennium Institute – Biodiversity of Antarctic and Sub‐Antarctic Ecosystems (BASE) Santiago Chile; ^8^ School of Biosciences University of Birmingham Birmingham UK; ^9^ Departamento de Microbiologia Universidade Federal de Minas Gerais Belo Horizonte MG Brazil

**Keywords:** cryptic diversity, King George Island, molecular assessment

## Abstract

The Antarctic Peninsula shows a fast‐rising temperature trend, increasing the risk of colonization by non‐native species, such as the invasive *Poa annua* . The “cryptic diversity” of largely unknown microscopic propagules in soil and snow poses a significant threat, as traditional morphological surveys are inadequate. Therefore, DNA metabarcoding of environmental DNA was applied to achieve a broader and more accurate assessment. We investigated non‐fungi eDNA diversity in soil (three sites) and freshly deposited snow (five sites) from Martel Inlet, King George Island, with sites categorized by proximity to human activity. DNA was extracted, and the internal transcribed spacer 2 (ITS2) was used as the DNA barcode. Sequencing was performed using an Illumina MiSeq V3. Sequences were classified into 72 ASVs, representing four kingdoms and 10 phyla, with 18% not previously recorded in Antarctica. Soils hosted richer communities (mean of 30 ASVs per sample) than snow (mean of 16.2 ASVs per sample); however, there was no statistically significant difference in abundance nor diversity between the sample types. The data did not reveal differences between the sampled areas or suggest anthropogenic influence on any of the sampled communities. This work underscores the value of eDNA metabarcoding in assessing cryptic biodiversity.

## 1 Introduction

Global warming has brought many negative impacts around the planet, with few places showing a faster rising temperature trend than the region of the Antarctic Peninsula (AP) (Convey 2001; Turner et al. 2005, 2016). This warming is leading to snow and ice melt, which is predicted to increase the extent of available ice‐free ground, creating new terrestrial habitats and increasing the availability of liquid water over the next century (Convey 2001; Pearce et al. 2009). These newly exposed soils, alongside seasonal snowpacks, act as the primary recipients for biological colonization, creating a landscape increasingly suitable for the establishment of new species (Galera et al. 2018; Union 2010).

This is a particular concern for King George Island (KGI), the most intensive focus of human activity in the South Shetlands Islands. Due to the proximity of this archipelago to South America and its relatively mild climate, KGI is one of the most likely places for non‐native species to be able to colonize (Chown et al. 2012). KGI hosts research and logistics facilities of 11 nations (Argentina, Brazil, Chile, China, Ecuador, Peru, Poland, Russia, South Korea, the United States of America, Uruguay). In addition, between 2019 and 2020, at least 73,156 tourists visited the AP region (http://www.iaato.org). This intense human presence provides as a major vector for the potential introduction of non‐native propagules (vegetative propagules, seeds, spores) directly into the Antarctic environment, in particular via clothing, equipment and cargo (Lityńska‐Zając et al. 2012; Benitez et al. 2024). Natural vectors such as wind and birds can also contribute to dispersal, particularly of microbial groups and microscopic propagules (Kappen and Straka 1988; Li et al. 2010; Pearce et al. 2010). However, current evidence suggests that anthropogenic transport by far outweighs that of natural processes (with no known examples of successful colonization of macroscopic organisms in the entire history of human contact with the continent), posing an unprecedented threat of biological invasion and jeopardizing Antarctica's unique native ecosystems (Rückamp et al. 2011; Pearce et al. 2009; Galera et al. 2018; Benitez et al. 2024).

Antarctica's native terrestrial flora is dominated by bryophytes and lichens, with only two native angiosperms, *Colobanthus quitensis* and *Deschampsia antarctica* . Likely introduced by expeditioners, the successful establishment of *Poa annua* in several locations and its invasive status in parts of Admiralty Bay on KGI already highlight the vulnerability of these ecosystems (Olech and Chwedorzewska 2011). However, the most immediate threat may not be from visible invaders but from the unseen propagules accumulating in the environment. The diversity of organisms arriving that possess the capability to remain dormant until suitable conditions for development occur. This kind of cryptic diversity remains largely unknown because of its capability of existing as spores, seeds, or microscopic fragments within soil and snow (Pearce et al. 2010; Rosa, Pinto, et al. 2020; Câmara, Carvalho‐Silva, et al. 2021; Carvalho‐Silva et al. 2021; Câmara et al. 2025). Furthermore, virtually no information exists relating to the dispersal or establishment of anthropogenically‐associated non‐native microbiota in Antarctica.

Until recently, Antarctic ecological surveys have largely relied on morphological analysis of established organisms, which demands specific taxonomic expertise and is not suitable for detecting cryptic organisms or propagules. Additionally, the morphology of some organisms in Antarctica can be directly influenced by environmental factors, resulting in phenotypic plasticity, as observed in the mosses *Roaldia revoluta* and various members of the genus *Bryum*, making morphological analysis a taxonomic tool that is not entirely reliable (Câmara, Valente, et al. 2019). Therefore, the use of molecular markers, such as DNA sequences, becomes essential for elucidating species diversity, as demonstrated in a re‐evaluation of the genus *Bartramia* where phylogenetic analyses revealed the presence of two species in Antarctica rather than one, as previously thought (Câmara, Soares, et al. 2019).

With the rapid developments, increasing availability, and reducing costs of sequencing technologies, tools such as DNA barcoding (Hebert et al. 2003; Hollingsworth et al. 2009) and DNA metabarcoding (Taberlet et al. 2012) can now be applied to achieve broader and more accurate biodiversity descriptions. By analyzing “environmental DNA” (eDNA) extracted directly from soil, water or snow, this technique allows the detection of a broad spectrum of organisms, including those that are rare, microscopic or taxonomically challenging, without first needing to isolate them (Fraser et al. 2018; Ruppert et al. 2019; Deiner et al. 2017; Rippin et al. 2018). Applying eDNA metabarcoding surveys to Antarctic soil and snow can provide unprecedented insight into the diversity of non‐fungal eukaryotes present (Rippin et al. 2018; Câmara et al. 2024; Rosa, da Silva, et al. 2020) and help understand the early stages of biological colonization or invasion.

In this study, we investigated the cryptic eDNA diversity present in snow and soil samples from Martel Inlet on KGI. We aimed to address the following questions: (1) which plants and other eukaryotic taxa are in the eDNA at this site's soil and snow? (2) Is the presence of assigned sequences of non‐native taxa associated with sites of high human activity? (3) Are species present among the assigned taxa that may have the potential to colonize the region under predicted climate and environmental changes?

## 2 Methods

### 2.1 Study Sites

**FIGURE 1:**
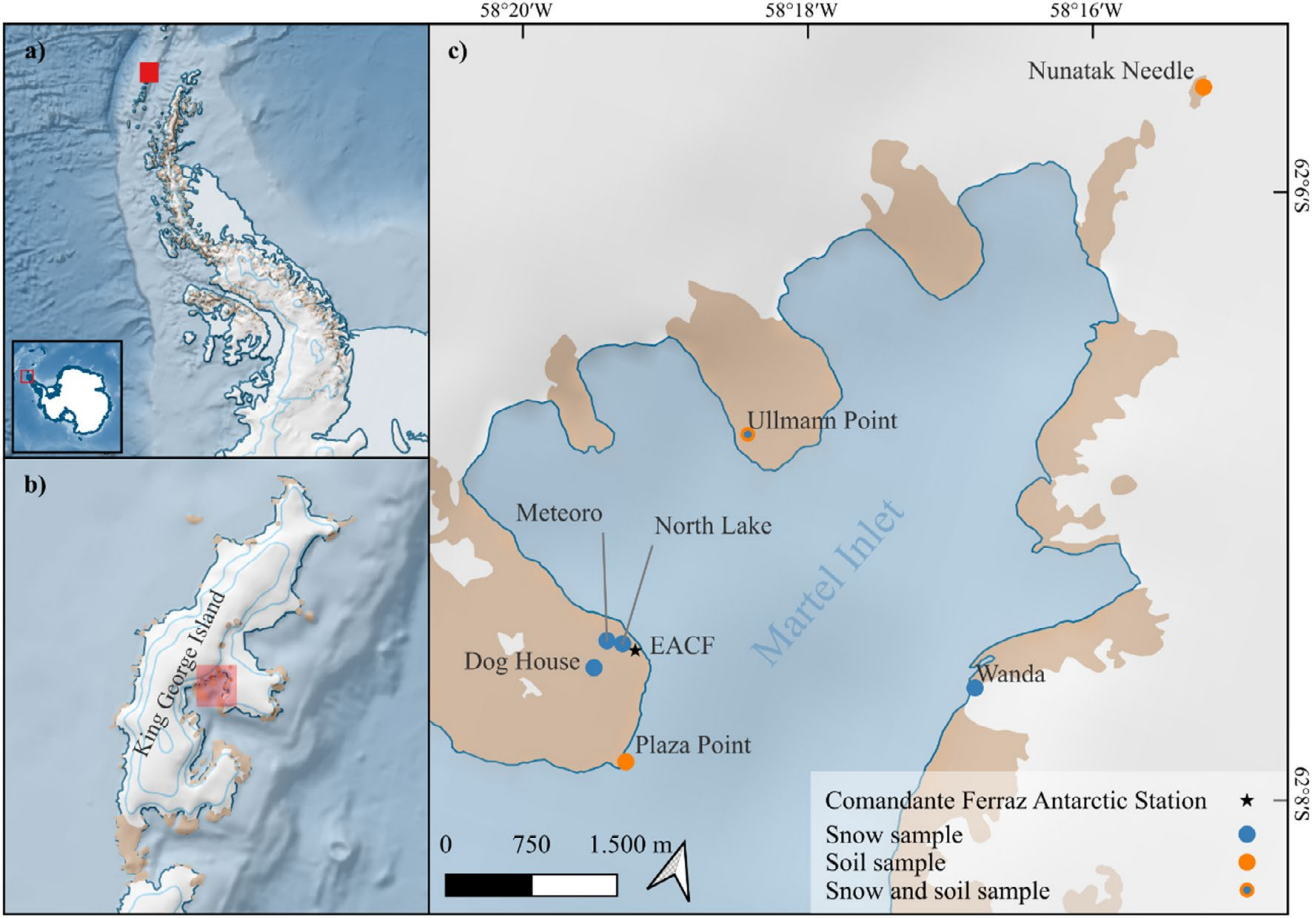
Study area. (a) Location of King George Island off the northern tip of the Antarctic Peninsula; (b) Location of Martel Inlet on King George Island; (c) Location of the Comandante Ferraz Antarctic Station (EACF) in Martel Inlet, showing locations of sampling sites.

Martel Inlet (Figure 1) is located in Admiralty Bay, the largest bay on King George Island (KGI), within Antarctic Specially Managed Area (ASMA) No. 1. The site, where the Brazilian Station Comandante Ferraz (EACF) is located, is relatively sheltered from the strong winds that typify the Bransfield Strait and Drake Passage, south and north of the island. Its microclimate is therefore distinct from the other parts of the island, characterized by an average annual temperature of around −1.8°C with mean seasonal temperatures ranging from approximately 0.9°C during summer (December–February) to −7°C during winter (June–August) (Ferron et al. 2004; ATCM document 2005). In addition to numerous scientists, supporting personnel, and research expeditions, Martel Inlet is also visited by tourists, the latter predominantly from ‘expedition’ cruise vessels along with a small number of private yachts (ATCM document 2005).

To evaluate the impact of human presence, we classified the samples from the seven sites based on their level of anthropization. Using the research team's local expertise, each site was designated as either more (+) or less (−) influenced by human activity, taking into account factors such as its proximity to research facilities and how often they are visited by scientists and even tourists (Table 1).

**TABLE 1:** Sample type and numbers of samples obtained from snow and soil at each sampling site in Martel Inlet, King George Island.

Sample kind	Dog house	Ullmann point	Wanda	North lake	Meteoro	Plaza point	Nunatak needle
Snow	3	3	1	1	1	0	0
Soil	0	3	0	0	0	3	3
Human presence	+	−	−	+	+	+	−

### 2.2 Soil Sampling

During the summer of 2020/2021, soil samples were collected from three locations: Plaza Point, Ullmann Point, and close to Nunatak Needle. At each location, three separate samples were taken (totaling nine samples) (Table 1), collected at least 50 cm apart. Each sample consisted of about 250 g of soil collected from the top 5 cm of the soil profile using a sterile spatula. Care was taken to avoid areas with visible vegetation. Immediately after collection, samples were sealed in sterile Whirl‐Pak plastic bags and stored at −20°C until DNA extraction took place.

### 2.3 Snow Sampling

During the summer of 2020/2021, freshly deposited snow samples were collected from five different sites: Dog House and Ullmann Point (three samples each), plus single samples from North Lake, Wanda, and Meteoro (Table 1). Approximately 2 L of snow was collected per sample using sterilized shovels and buckets. The snow was allowed to melt at room temperature in the microbiology laboratory at EACF (Comandante Ferraz Antarctic Station) and was then filtered through 0.22 μm Sterivex filters using a Millipore chemical pump system to capture microbial content. All filtration procedures were conducted under sterile conditions in a laminar flow chamber to prevent contamination. The Sterivex filters were then frozen at −20°C until DNA extraction.

### 2.4 DNA Extraction and Amplification

DNA was extracted from the soil samples using the FastDNA SPIN Kit for Soil (MP Biomedicals, OH, USA). For the thawed snow samples, we used the DNeasy PowerWater Sterivex Kit (QIAGEN/Düsseldorf, Germany). In both cases, the manufacturer's instructions were followed. Extractions were carried out under strict sterile conditions. DNA quality was analyzed using agarose gel electrophoresis (1% agarose in 1 × Trisborate‐EDTA) and then quantified using the Quanti‐iT Pico Green dsDNA Assay (Invitrogen). Extracted DNA was used as a template for generating PCR amplicons. The internal transcribed spacer 2 (ITS2) of the nuclear ribosomal DNA was used as a DNA barcode for molecular species identification as it has been used for identifying a diverse range of eukaryote organisms including fungi, animals, protozoans, chromists, and plants, and also proved effective in the last studies regarding Antarctic diversity (Chen et al. 2010; Richardson et al. 2015; Rosa, da Silva, et al. 2020; Rosa, Pinto, et al. 2020; Ruppert et al. 2019). The PCR amplicons were generated using the universal primers ITS3 and ITS4 (White et al. 1990). Library preparation and high‐throughput sequencing were performed using an Illumina MiSeq V3 (Illumina Inc.) at Macrogen Inc. (South Korea), where high‐quality fragments of approx. 600 bp (2 × 300) were obtained.

To monitor for potential contamination during the laboratory procedures, negative controls were included during both DNA extraction (blank extraction) and PCR amplification (no‐template control, NTC). These controls were processed alongside the environmental samples using the same kits and protocols. No visible amplification was observed in the negative controls via agarose gel electrophoresis, and they yielded no detectable sequencing reads after bioinformatic processing, confirming the integrity of the results.

### 2.5 Data Analyses

The raw fastq files were filtered and cleaned using BBDuk version 38.34 (BBMap—BUSHNELL B. ‐ sourceforge.net/projects/bbmap/), where sequences with quality < 30 (Phred Score) and/or number of base pairs < 75 were discarded. The remaining sequences were imported into the program QIIME2 version 2026.1 (https://qiime2.org/) for bioinformatics analysis (Bolyen et al. 2019). The qiime2‐dada2 plugin is a complete pipeline that was used in additional replication filtering, “paired‐end” sequence joining, and chimera removal (Callahan et al. 2016). Using the qiime2‐feature‐classifier command, “amplicon sequence variants” (ASVs) were generated, and these were compared via the classify‐sklearn command against the PLANiTS2 database (Banchi et al. 2020), the latest version. ASVs classified as unknown or at a level lower than the taxonomic family classification were filtered and compared with the UNITE ITS all eukaryotes version 10.0 database (Abarenkov et al. 2020), the latest version. Finally, ASVs classified as unknown or at a level lower than the family taxonomic classification were filtered and contrasted against the NCBI nonredundant nucleotide sequences (nt) database (May 2026) using BLASTn software (Camacho et al. 2009) with the default parameter. Taxonomic assignments of the identified ASVs were achieved using the MEGAN6 software using the (Huson et al. 2016) which applied the Lowest Common Ancestor (LCA) algorithm to resolve BLAST alignments and generate the final taxonomic abundance matrix. The number of sequences for each taxon was used as a measure of abundance following Deiner et al. (2017), Câmara, Carvalho‐Silva, et al. (2021), Hering et al. (2018) and Câmara et al. (2024). Classification and systematics for kingdoms and phyla followed Ruggiero et al. (2015). For minor taxonomic classifications, Worms Editorial Board (Horton et al. 2022), Guiry and Guiry (2022), and Bánki et al. (2025) were consulted and, for geographical distribution, Thompson et al. (2019) and Guiry and Guiry (2022) and Tropicos (www.tropicos.org) were consulted. Rarefaction curves were calculated with the classified ASVs from each sample with the iNEXT package (iNterpolation and EXTrapolation) (Hsieh et al. 2016). Non‐metric multidimensional scaling (NMDS) was used to visualize the relationship between samples, ASVs composition, and human presence. The permutational multivariate ANOVA (PERMANOVA) test based on 999 permutations, using the adonis2 function in the Vegan package (Oksanen et al. 2022) in the R statistical environment (Conway et al. 2017), was used to analyze the influence of human presence on the assigned community composition. For the NMDS, Euclidean distance on Hellinger‐transformed data was used (Legendre and Gallagher 2001). Species diversity and richness for each site were assessed by calculating the Simpson, Fisher's alpha, and Margalef indices. In addition, a diversity *t‐*test was performed for comparison of the Shannon and Simpson diversities between samples, sample sites, and sample types. To analyze and visualize the distribution of shared and exclusive ASVs among the sample types, an UpSet plot was generated using the UpSetR package in the R statistical environment (Conway et al. 2017).

## 3 Results

A total of 1,625,695 ITS2 paired‐end DNA reads were generated during sequencing. After quality filtering and cleaning of the raw data, 1,277,968 (78%) sequences remained for taxonomic classification. The rarefaction curves calculated for all the samples collected reached an asymptote (Figure S1), indicating that the sampling effort was sufficient, and the analyzed sequences provide a good representation of the sequence diversity present in the sampled substrates. These sequences were subsequently dereplicated and processed to infer amplicon sequence variants (ASVs).

**FIGURE 2:**
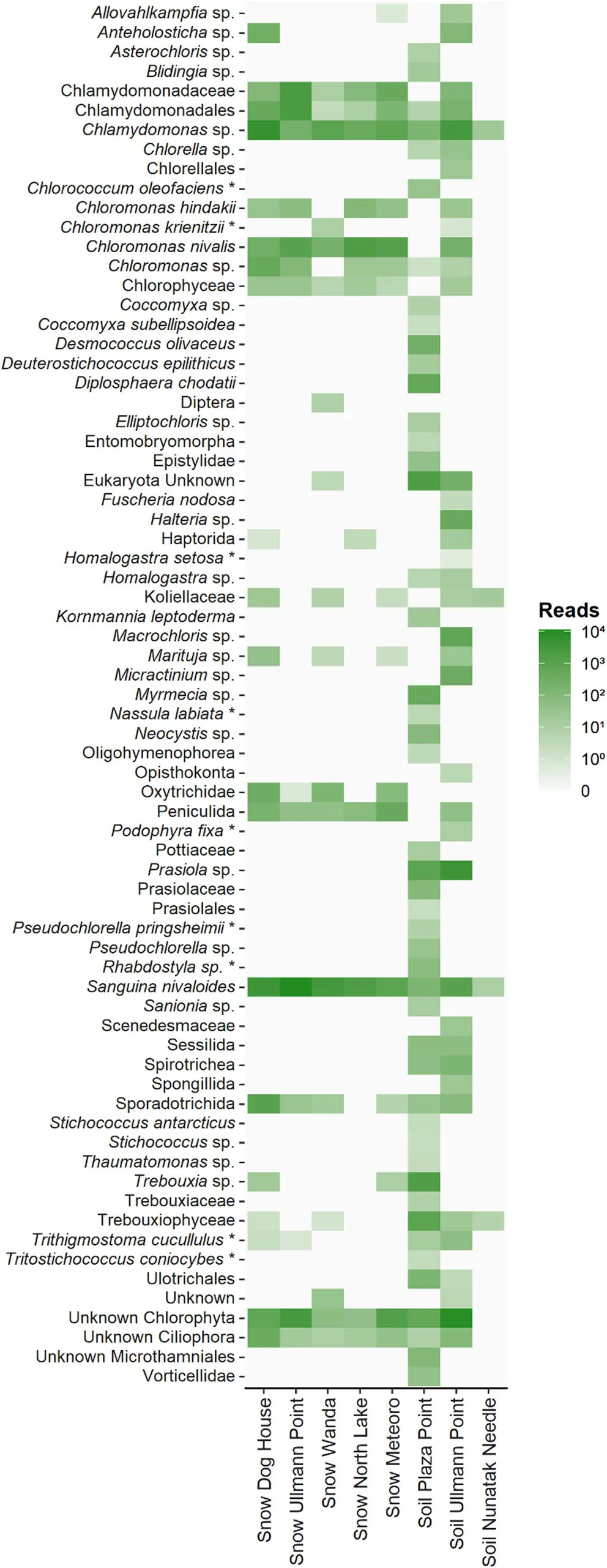
Abundance of taxa assigned from eDNA samples obtained on King George Island. Taxa not previously recorded from Antarctica are indicated by *.

After taxonomic classification and discarding of sequences identified as fungi (500,442 or 39%), already reported by Rosa et al. (2021, 2022), the remaining 777,526 (61%) sequences were classified into 72 ASVs, with < 1% of sequences being classified as unknown (Table S1, Figure S2, Figure 2).

The classified ASVs included representatives of four kingdoms (Animalia, Chromista, Viridiplantae, Protozoa) and 10 phyla (Arthropoda, Cercozoa, Chlorophyta, Chordata, Ciliophora, Cnidaria, Mollusca, Percolozoa, Porifera, Streptophyta). Of the 72 ASVs assigned, 18 (25%) were assigned to species level (Table S2, Figure 2), and 13 (18%) have not been previously recorded in Antarctica.

Among the unknown ASVs, 367 reads could not be assigned by the three datasets consulted. Unknown Eukaryota represented 2.6% of the sequences obtained (20,067 reads), and 38 reads were associated with the Opisthokonta, which include all animals and fungi.

**FIGURE 3:**
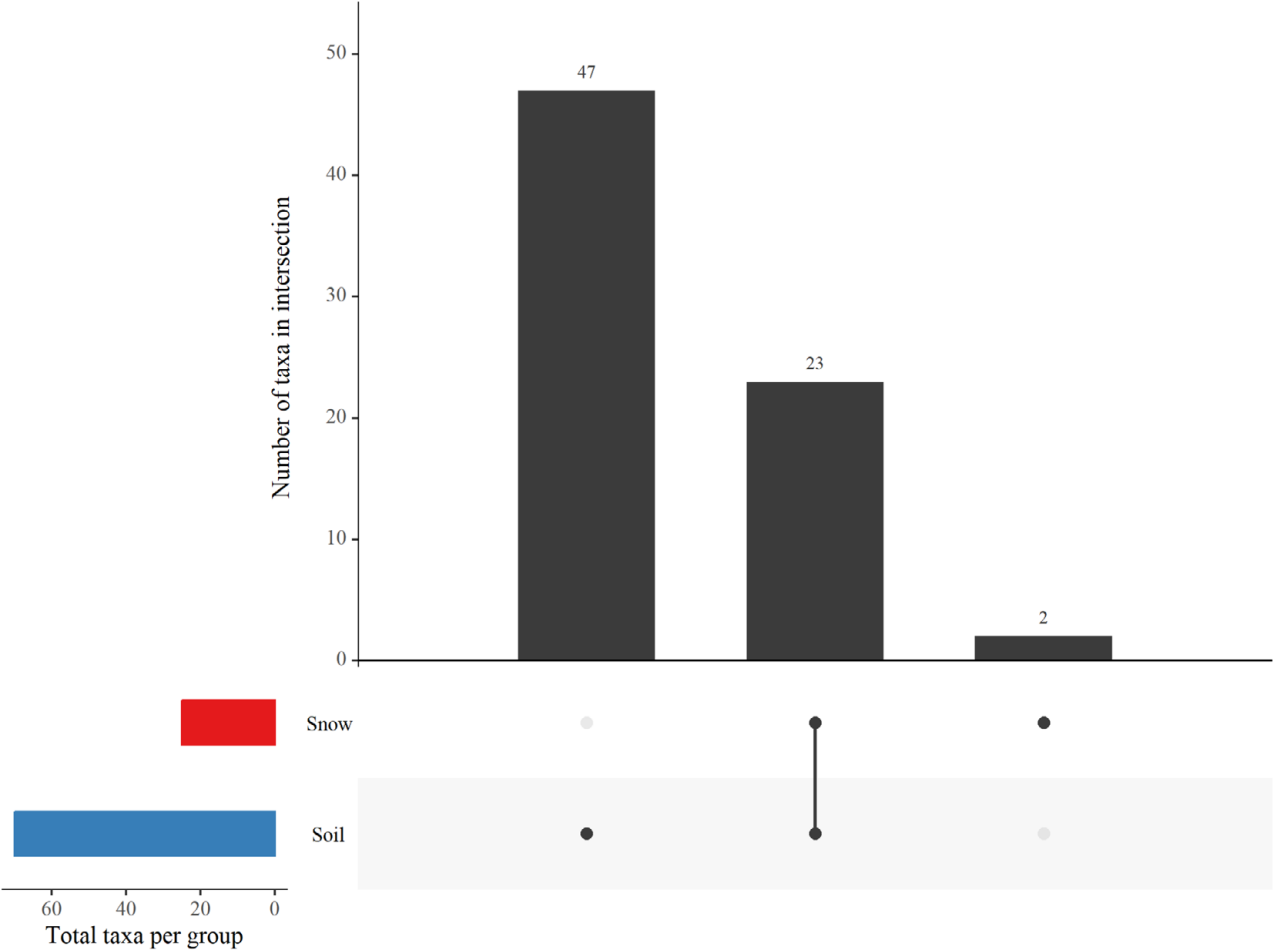
UpSet plot showing the number of assigned taxa found uniquely within and shared between soil and snow samples collected at Martel Inlet, King George Island.

**FIGURE 4:**
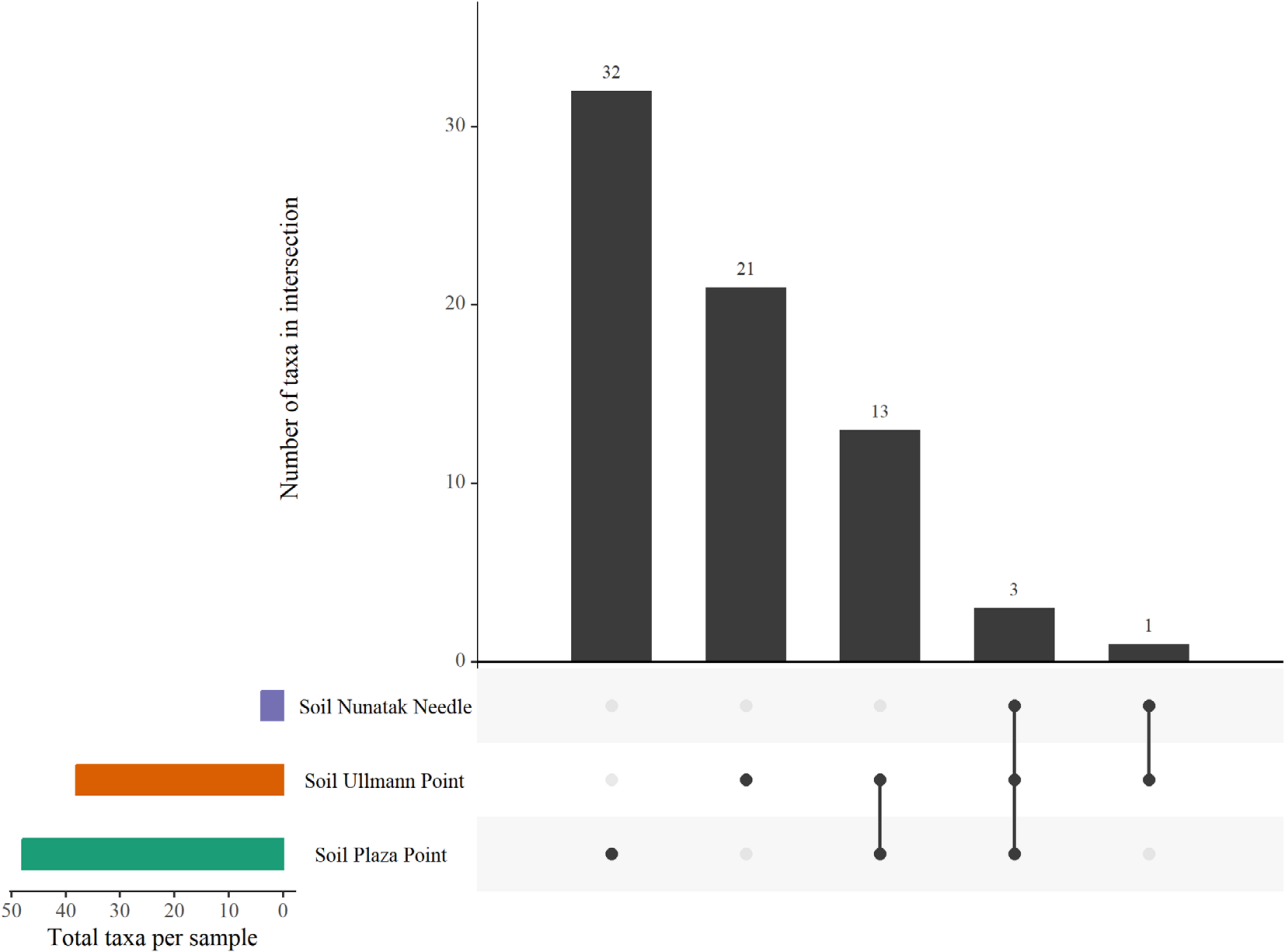
UpSet plot showing the numbers of assigned taxa found uniquely in and shared between each of the soil sampling sites in Martel Inlet, King George Island.

**FIGURE 5:**
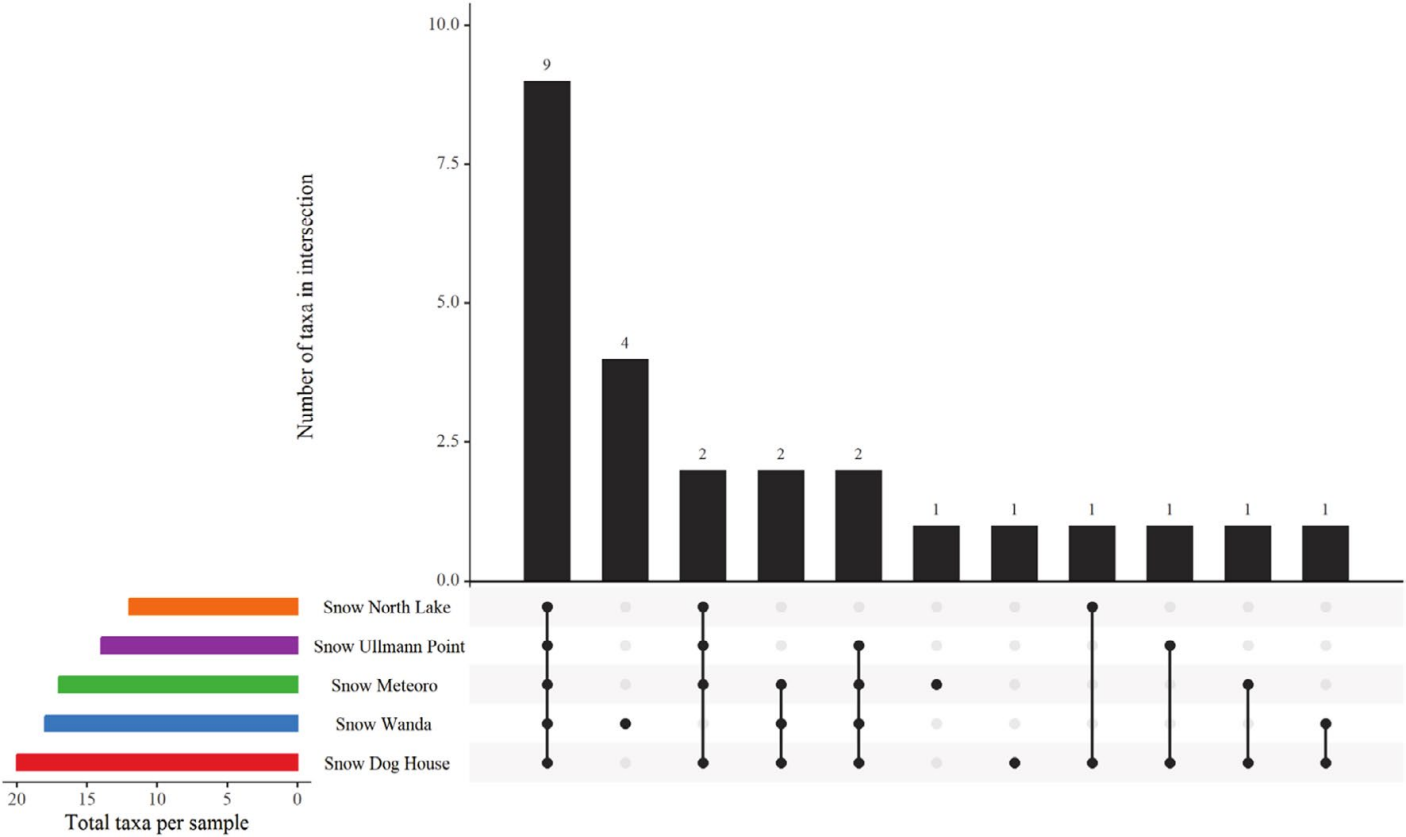
UpSet plot showing the numbers of assigned taxa present uniquely in and shared between each of the snow sampling sites in Martel Inlet, King George Island.

The first UpSet plot illustrates the distribution of the 72 distinct assigned ASVs across the snow and soil substrates, highlighting a core community of 23 ASVs that were shared between them (Figure 3). Soil samples were the richest, containing 70 assigned ASVs in total, of which 47 were found only in this substrate (Figure 3). In contrast, the snow environment hosted 25 ASVs, with only two being unique to it (Figure 3). Notably, despite the lower taxonomic richness, the snow samples generated a majority of sequencing reads (62%; 483,375), indicating a community dominated by few organisms. When evaluating diversity per location, the soil sampling sites exhibited considerable variation in ASV distribution; only three taxa were shared across all soil locations, while Plaza Point hosted 32 exclusive taxa and shared the majority of its remaining ASVs with Ullmann Point (Figure 4). Conversely, taxa were more evenly distributed across the snow sampling sites, with nine ASVs shared among all locations. Within the snow group, the Wanda site presented the highest number of unique taxa, containing four exclusive ASVs (Figure 5).

The Viridiplantae was the richest and most abundant group found in the samples, accounting for around 91% of the total reads, with unknown Chlorophyta (green algae) alone representing 20% of the total sequences. *Sanguina nivaloides* Procházková, Leya, Krížková, & Nedbalová 2019 (Chlorophyta) was the most abundant assigned taxon and was present in all samples, followed by unidentified Chlorophyta and the genus *Chlamydomonas*.

As shown in Table 2, the soil from Plaza Point hosted the richest assigned diversity (Simpson's index), while that from Nunatak Needle was the least diverse, with only 4 taxa. Snow sampled from Wanda contained the lowest diversity among all samples. Overall, there were no significant differences in diversity or richness between the assigned communities from snow and soil samples (*t*‐test, *p* > 0.05).

**TABLE 2:** Diversity indices of assigned ASVs present in soil and snow samples obtained in Martel Inlet, King George Island.

Sample	ASVs richness	Simpson	Fisher's *α*	Margalef
Snow Dog House	20	0.75	1.77	1.60
Snow Ullmann Point	14	0.63	1.16	1.06
Snow Wanda	18	0.52	1.78	1.59
Snow North Lake	12	0.65	1.13	1.03
Snow Meteoro	17	0.83	1.63	1.46
Soil Plaza Point	48	0.88	4.92	4.14
Soil Ullmann Point	38	0.72	3.45	3.02
Soil Nunatak Needle	4	0.70	0.59	0.48

**FIGURE 6:**
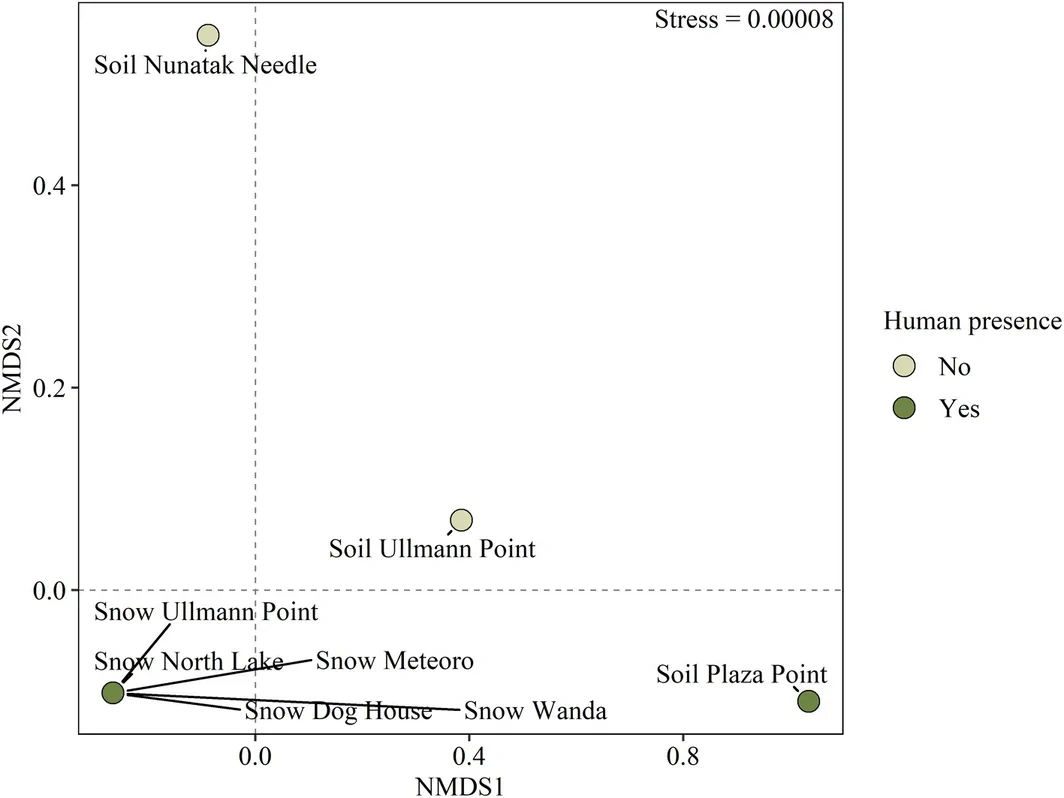
Non‐metric multidimensional scaling (NMDS) analysis based on the Euclidean distance of community composition assigned from soil and snow samples collected on King George Island, Antarctica (based on molecular ASV abundances). Relative abundance data were Hellinger‐transformed. Samples are colored according to the influence of human presence at the site.

The NMDS plot (Figure 6) illustrates that the stronger human influence showed no obvious link with the assigned communities present in the different samples, also supported by the nonsignificant PERMANOVA.

## 4 Discussion

First, it is important to remember that a detected DNA sequence does not confirm the presence or viability of a given organism, and targeted studies are required to confirm the presence of a species. Our data suggest that the KGI soils examined hosted richer and more diverse assigned communities, with a mean of 30 ASVs per sample, in comparison with only 16.2 ASVs per snow sample; however, there was no overall statistically significant difference in abundance or diversity between the sample types. The overlap observed between the snow and soil communities, with 23 shared ASVs of the 72 assigned, indicates a link between the snow and soil communities. Closer examination of these shared ASVs indicates that they form a core snow‐terrestrial community, dominated primarily by photosynthetic algae representing the phylum Chlorophyta (e.g., *Chlamydomonas* sp., *Sanguina nivaloides*, *Chloromonas nivalis (Chodat) Hoham & Mullet*, 1978) and their primary consumers, various protozoans representing the phylum Ciliophora. This core community may be linked through snowmelt depositing its microbial community into the underlying soil, which may then, in turn, serve as a seasonal reservoir. The soil community's complexity is highlighted by the presence of 47 exclusive ASVs, including representatives of purely terrestrial groups such as Arthropoda and Bryophyta. The detection patterns of Diptera and Oxytrichidae sequences, exclusive to the snow layer, reveal distinct ecological phenomena. Although winter gnats such as *Trichocera maculipennis* , which are widely distributed across surrounding ice‐free terrestrial sites in Maritime Antarctica, are well‐documented for their high cold‐tolerance and active locomotion over snowpacks for dispersal or mating behaviors (Chwedorzewska et al. 2013; Hagvar and Krzemin 2007), the presence of Diptera sequences in a single snow sample suggests a transient biological trace rather than an established population. In contrast, sequences belonging to the ciliate family Oxytrichidae were consistently detected across all but one analyzed snow sample. This distribution indicates a stable and widespread biological presence within the snowpack. Certain members of Oxytrichidae are recognized as highly adapted, cold‐tolerant micro‐predators capable of maintaining active metabolic and feeding roles (primarily preying on bacteria) within snow layers and cryogenic micro‐environments (Felip et al. 1995).

Studies such as those of Carvalho‐Silva et al. (2021), who reported eDNA assignments of 16 streptophytes ASVs in soil from Deception Island, and Câmara, Carvalho‐Silva, et al. (2021), who recorded 65 chlorophyte assignments on the same island, give some context to the current study, although Deception Island is an environment influenced by exclusive conditions due to its volcanic nature. Combined, these two studies reported 81 Viridiplantae ASVs, while in the current study, we found a lower total of 43, 11 being shared between studies.

Multiple reasons could underlie these differences, most notably the stark contrast in the intensity and nature of human presence. Deception Island is historically one of the most heavily visited areas in the Antarctic Treaty region, acting as a major hub for commercial tourism with thousands of passenger landings annually, alongside its legacy of industrial whaling (Roura 2012). This massive influx of tourist traffic significantly increases the risk of accidental biointroductions and propagule dispersal via clothing, footwear, and equipment, which are often less strictly controlled than on dedicated scientific expeditions. This continuous anthropogenic pressure may favor the introduction and detection of transient or cosmopolitan micro‐eukaryotes, inflating local ASV richness. In contrast, the areas analyzed in our current study experience a much lower, strictly regulated, and highly localized footprint, restricted almost entirely to transient scientific activities. Additionally, the existence of a more diverse range of habitats and associated Viridiplantae communities at Deception Island is strongly influenced by its active geothermal nature (Smith 2005), further contributing to the higher diversity observed there.

In the current study, the soil sampled at Plaza Point had the highest richness and diversity, followed by that at Ullmann Point and Nunatak Needle. Although Plaza Point is closest to a center of human activity, no overall anthropogenic influence on diversity was apparent in our data, and this trend may be linked to the geographical locations of the sampling sites. All the sampled sites are located close to the coast, but their specific positions in Martel Inlet may drive community differences. For instance, Plaza Point is the site closest to the entrance of the inlet. Its constant exposure to strong winds and marine spray from the Bransfield Strait may make a significant contribution to the community composition, with 32 assigned ASVs exclusively present at this location. This marine influence is further supported by the presence of several marine ASVs in Plaza Point soil, such as *Thaumatomonas* sp. and *Nassula labiata* Kahl, 1933 and representatives of Ulvophyaceae, which were not detected from the other sampling sites.

The non‐fungal eukaryotic diversity assigned from the snow samples obtained in Martel Inlet is consistent with patterns reported from other locations in the Maritime Antarctic. The community composition, dominated by algae representing the phylum Chlorophyta, is consistent with descriptions of algal blooms on the Fildes Peninsula, also on KGI (Soto et al. 2020) and with algal communities studied further south in Ryder Bay (Davey et al. 2019). More broadly, the taxonomic profile resembles that found in a regional study across the Antarctic Peninsula, which identified Trebouxiophyceae and *Chlamydomonas nivalis* as the most abundant ASVs in various snow samples (Câmara et al. 2024). However, we recorded between 12 (North Lake) and 20 (Dog House) assigned ASVs per sampling location, a somewhat higher value than reported in a similar study on Livingston Island, where a maximum of 10 ASVs were reported in a single snow sample (Câmara et al. 2025).

Representatives of the kingdom Viridiplantae formed the richest and most abundant group across all samples. The ITS2 region is particularly effective at providing high taxonomic resolution for several green algal lineages, although the resolution achieved varies among groups depending on marker variability and reference database completeness, which may have contributed to the higher taxonomic resolution for this phylum over other eukaryotic lineages. Most records represented the green algal classes Trebouxiophyceae, Chlorophyceae, and Ulvophyceae, which are found in diverse terrestrial and aquatic environments in the form of coccoid to ellipsoid unicells, filaments, blades, and colony‐forming organisms (Muggia et al. 2018). In this study, the Trebouxiophyceae contributed 22 ASVs, followed by Chlorophyceae with 14 ASVs and the majority of individual sequences (59%), and the Ulvophyceae with two ASVs, *Kornmannia leptoderma* (Kjellman) Bliding and *Blidingia* sp. (Leliaert et al. 2012).

Among the Chlorophyceae, *Sanguina nivaloides* was the most abundant taxon detected in the present study and occurred in all samples. This species has a cosmopolitan distribution in regions with perennial snow and is associated with the “blood snow” phenomenon (Procházková et al. 2019). Representatives of the genus *Chloromonas* are also associated with this phenomenon and can be abundant in samples enriched by animal‐derived nutrients (Remias et al. 2013). Our data include several records of ASVs new to Antarctica. However, we recognize that eDNA sequence assignments are limited by the completeness of the databases available, and do not unequivocally confirm the presence or viability of a given taxon. Some representatives of the very abundant genus *Chlamydomonas* (13.46% of total DNA reads) are well‐known and widespread snow algae also occurring in freshwater/terrestrial habitats (Guiry and Guiry 2022; Stibal et al. 2007). In this study *Chlorococcum oleofaciens* Trainor & H.C. Bold, 1954 and *Chloromonas krienitzii* Matsuzaki & Nozaki, 2015 represented new records for Antarctica, whereas the family Scenedesmaceae represented a new record for KGI. Several Viridiplantae ASVs assigned in this study have previously been recorded only in cold environments of the Northern Hemisphere. Their presence can be interpreted through two primary hypotheses rather than assuming recent introduction. The first is that they represent cryptic native species: Antarctic lineages that could appear morphologically and genetically similar to Northern Hemisphere populations, but have evolved in isolation, and the ITS2 region will not be able to differentiate between them. The second hypothesis is that these ASVs have a natural bipolar distribution, resulting from effective long‐distance dispersal over evolutionary time, which would define their presence in Antarctica as part of a long‐established natural range.

The most abundantly assigned taxon in the class Trebouxiophyceae was *Prasiola* sp. Members of this genus are commonly found growing as macroscopic foliose thalli in nutrient‐enriched areas in Antarctica, as well as in lichenized form (*Mastodia tessellata*). *Prasiola crispa* (Kützing) Knebel can be extremely common in ice‐free areas of maritime Antarctica (Vinocur and Pizarro 1995, 2000; Kovácik and Pereira 2001), and the genus has been reported in several metabarcoding studies conducted in the region (Fonseca et al. 2022; Câmara et al. 2024; Câmara, Carvalho‐Silva, et al. 2021; Câmara et al. 2025). Several members of this class represented new regional records. *Pseudochlorella pringsheimii* (Shihar & Krauss) Darienko & al., 2010 and *Tritostichococcus coniocybes* (Letellier) Pröschold & Darienko, 2020 were recorded for the first time in Antarctica, whereas *Desmococcus olivaceus* (Persoon ex Acharius) J.R. Laundon, 1985 and *Deuterostichococcus epilithicus* Pröschold & Darienko, 2020 were recorded for the first time on KGI.

Trebouxiophyceae ASVs found here included organisms such as the well‐known genus *Chlorella*, members of which have been widely used as model organisms for pioneering plant physiological and biochemical studies and used in agriculture and biotechnology research (Huss et al. 1999), as well as other typically planktonic ASVs such as *Micractinium*. ASVs assigned to the genus *Blidingia* and species *Kornmannia leptoderma* are the first records of these taxa from KGI. The high abundance of Trebouxiophyceae at the studied sites could be linked to the presence of the fungal genera *Hypogymnia*, *Lecidea*, and *Placopsis*, which were detected by Rosa et al. (2022). Given that these fungi are common symbionts for *Trebouxia*, it is highly probable that this algal family is represented by lichenized forms in these environments.

The second most abundant phylum of Viridiplantae, Streptophyta, was represented by *Sanionia* sp., and one member of the family Pottiaceae, together representing around 0.03% of the total reads. *Sanionia* is one of the most widespread moss genera in this region, forming the major component of extensive moss carpets found on low‐lying ground on KGI (Câmara, Carvalho‐Silva, et al. 2021; Câmara, Convey, et al. 2021) as well as throughout the Maritime Antarctic. Pottiaceae is the second largest Antarctic moss family with 17 species, most of which are rare and inconspicuous, although some ASVs, such as *Syntrichia* sp., for example, are also widespread and can form significant carpets around KGI and more widely in the Maritime Antarctic.

Chromista was the second most abundant and diverse kingdom, with almost 6% of reads, and was present in all samples except soil from Nunatak Needle. This kingdom was predominantly represented by the phylum Ciliophora, a major group of bacterivores and micrograzers that play a crucial role in the microbial loop by channeling energy from bacteria to higher trophic levels (Cavalier‐Smith 2018). Within Ciliophora, the class Oligohymenophorea was the most abundant and richest, followed by the order Peniculida. The order Sessilidae was represented in our samples by new records for King George Island (KGI), such as the family Epistylididae and the genus *Rhabdostyla* sp., both known as sessile epibionts, as well as the freshwater and marine species *Marituja* sp. (Hu et al. 2019; Souza et al. 2024). Additionally, the class Spirotrichea, represented here by the order Sporadotrichida, was present in almost all samples, highlighting the widespread distribution of these active microbial predators in our studied samples.

Other ciliates detected in this study represent new assignments for the Antarctic continent, including the widespread freshwater and marine species *Nassula labiata*, *Homalogastra setosa* Kahl, 1926, and *Trithigmostoma cucullulus* Jankowski, 1967, the latter usually associated with estuarine or high‐nutrient environments (Balczon and Pratt 1996). We also recorded the Arctic suctorian *Podophrya fixa* O. F. Müller, 1786, another epibiont whose presence suggests a bipolar distribution for these cold‐adapted *microeukaryotes*. Beyond ciliates, just another phylum of Chromista was detected, represented by a single taxon: Cercozoa, represented by the marine flagellated genus *Thaumatomonas* De Saedeleer, 1931.

The order Diptera was detected in a single snow sample from Wanda. Although Antarctica has only two native insect species, the midges *Belgica antarctica* Jacobs, 1900 and *Parochlus steinenii* (Gercke, 1889), increasing attention has been paid to the detection of alien insects in anthropized areas such as research stations (Chown and Convey 2016; Hughes et al. 2005). However, Wanda is a relatively pristine site with minimal human influence, making an established non‐native population unlikely.

A representative of the order Spongillida, a marine group, was recorded in one soil sample, again likely a result of proximity to the coast and exposure to marine spray. At Plaza Point, a representative of the ant genus *Myrmecia* was assigned from the soil samples. There are no native species of ant present in Antarctica, and no formal or colloquial records of this group. However, this location is a site of human activity, including a Brazilian refuge hut which is used as a simple research facility during the summer months, and various ants have been identified in recent horizon scanning studies that have included the Maritime Antarctic and sub‐Antarctic South Georgia (Hughes et al. 2020). Thus, any putative indication of the presence of ants in this region should raise concerns and highlight the importance of both effective biosecurity procedures and awareness, and careful monitoring of the sites of putative reports.

Finally, the kingdom Protozoa was represented by a single taxon. This taxon, *Allovahlkampfia* sp., was present in both soil and snow samples and is known from Antarctica (Tyml et al. 2016). Recent studies have shown that this amoeba may have environmental and clinical implications since it may act as a replicative host for several pathogenic bacteria (Mohamed and Huseein 2016).

## 5 Conclusions

In conclusion, our eDNA metabarcoding analyses of soil and snow samples from Martel Inlet revealed a rich cryptic biodiversity, primarily comprising microalgae and other protists, along with contributions from native Antarctic bryophytes. Despite our relatively limited sampling effort, our data did not indicate clear differences between the sampled areas, which were all within a relatively small geographical scale, nor did they suggest any strong anthropogenic influence on any of the sampled communities. Also, although only a few taxa were identified as new to Antarctica or the Antarctic Peninsula, fortunately, none were considered exotic invasive species that could threaten the studied environment. Our data support the value of applying DNA metabarcoding as a tool to assess the diversity and richness of ASVs present in Antarctic snow and soil. As is a feature of many such studies, the number of sequences not possible to assign to species level is indicative of the need to improve the coverage of sequence databases, particularly in the context of these cryptic taxa, and highlights the substantial gaps that remain in our knowledge of these elements of Antarctic biodiversity.

## Funding

This work was supported by Conselho Nacional de Desenvolvimento Científico e Tecnológico.

## Supporting information


**Figure S1:** Rarefaction curves by individual samples from air and snow collected at Martel Inlet on King George Island, Antarctica. (a) Rarefaction curves by individual samples (b) Rarefaction Curve by Individual Sample (Zoomed In to 2000 Reads).
**Table S1:** The number of sequences present in the samples obtained from Admiralty Bay on King George Island after applying the quality filters, and the number of sequences classified by each database.
**Figure S2:** Representation of the number of DNA sequences in each step of the classification of the snow and soil samples obtained from Admiralty Bay on King George Island.
**Table S2:** Abundance (number of DNA sequences) of taxa present in samples obtained on King George Island (classification according to Ruggiero et al. 2015). (*) indicates species not previously recorded for Antarctica.

## Data Availability

The data that support the findings of this study are available from the corresponding author upon reasonable request.
