# Supplementary material for: Non‐Fungi Diversity Present in Soil and Snow Impacted by Anthropogenic Activity in King George Island, Maritime Antarctica Using Metabarcoding Approach
Source: J Eukaryot Microbiol. 2026 Sep 11;73(5):e70122. doi: 10.1111/jeu.70122 (PMC13569865; doi:10.1111/jeu.70122)
Supplement: Supplementary file 1 [file JEU-73-e70122-s001.docx]

Supplementary Figure 1**.** Rarefaction curves by individual samples from air and snow collected at Martel Inlet on King George Island, Antarctica. a) Rarefaction curves by individual samples b) Rarefaction Curve by Individual Sample (Zoomed In to 2000 Reads).

a)

b)

b)

a)

Supplementary Table I**.** The number of sequences present in the samples obtained from Admiralty Bay on King George Island after applying the quality filters, and the number of sequences classified by each database.

| **SAMPLES** | **Raw sequences** | **Cleaned and Filtered seq** | **Class. by PlanITS** | **Class. by UNITE** | **Class. by Blast** | **Fungi UNITE** | **Fungi Blast** | **Unknown** |
| --- | --- | --- | --- | --- | --- | --- | --- | --- |
| **SNOW DOG HOUSE** | 328085 | 254389 | 56866 | 3633 | 79782 | 108880 | 5228 | 0 |
| **SNOW ULLMANN POINT** | 290298 | 226712 | 21524 | 6 | 178358 | 20498 | 6326 | 0 |
| **SNOW WANDA** | 92160 | 74211 | 9142 | 1418 | 32287 | 22900 | 8139 | 325 |
| **SNOW NORTH LAKE** | 73868 | 53426 | 4246 | 0 | 39931 | 3253 | 5996 | 0 |
| **SNOW METEORO** | 84783 | 66441 | 8443 | 827 | 46587 | 9413 | 1171 | 0 |
| **SOIL PLAZA POINT** | 324006 | 247758 | 26278 | 492 | 58092 | 161545 | 1351 | 0 |
| **SOIL ULLMANN POINT** | 299645 | 242565 | 31278 | 11503 | 165956 | 31705 | 2081 | 42 |
| **SOIL NUNATAK NEEDLE** | 132850 | 112466 | 354 | 0 | 156 | 111926 | 30 | 0 |
| **TOTAL** | 1625695 | 1277968 | 158131 | 17879 | 601149 | 470120 | 30322 | 367 |

Supplementary Figure 2**.** Representation of the number of DNA sequences in each step of the classification of the snow and soil samples obtained from Admiralty Bay on King George Island.

Supplementary Table II**.** Abundance (number of DNA sequences) of taxa present in samples obtained on King George Island (classification according to Ruggiero et al. 2015). (*) indicates species not previously recorded for Antarctica.

| **TAXA** | **SNOW DOG HOUSE** | **SNOW ULLMANN**  **POINT** | **SNOW WANDA** | **SNOW**  **NORTH LAKE** | **SNOW METEORO** | **SOIL PLAZA POINT** | **SOIL ULLMAN POINT** | **SOIL NUNATAK** |  |
| --- | --- | --- | --- | --- | --- | --- | --- | --- | --- |
| **ANIMALIA** |  |  |  |  |  |  |  |  | |
| **ARTHROPODA** |  |  |  |  |  |  |  |  | |
| Entomobryomorpha | 0 | 0 | 0 | 0 | 0 | 38 | 0 | 0 |  |
| Diptera | 0 | 0 | 84 | 0 | 0 | 0 | 0 | 0 |  |
| *Myrmecia* sp. | 0 | 0 | 0 | 0 | 0 | 30 | 0 | 0 |  |
| **PORIFERA** |  |  |  |  |  |  |  |  |  |
| Spongillida | 0 | 0 | 0 | 0 | 0 | 0 | 233 | 0 |  |
| **CHROMISTA** |  |  |  |  |  |  |  |  |  |
| **CERCOZOA** |  |  |  |  |  |  |  |  |  |
| *Thaumatomonas* sp. | 0 | 0 | 0 | 0 | 0 | 23 | 0 | 0 |  |
| **CILIOPHORA** |  |  |  |  |  |  |  |  |  |
| Unknown Ciliophora | 4134 | 171 | 100 | 145 | 421 | 80 | 1025 | 0 |  |
| Haptorida | 8 | 0 | 0 | 29 | 0 | 0 | 160 | 0 |  |
| *Fuscheria nodosa* | 0 | 0 | 0 | 0 | 0 | 0 | 27 | 0 |  |
| *Nassula labiata ** | 0 | 0 | 0 | 0 | 0 | 38 | 0 | 0 |  |
| Oligohymenophorea | 0 | 0 | 0 | 0 | 0 | 33 | 0 | 0 |  |
| Peniculida | 1852 | 476 | 453 | 779 | 4400 | 0 | 464 | 0 |  |
| *Marituja* sp. | 413 | 0 | 37 | 0 | 16 | 0 | 256 | 0 |  |
| *Homalogastra* sp. | 0 | 0 | 0 | 0 | 0 | 46 | 133 | 0 |  |
| *Homalogastra setosa ** | 0 | 0 | 0 | 0 | 0 | 0 | 4 | 0 |  |
| Sessilida | 0 | 0 | 0 | 0 | 0 | 638 | 585 | 0 |  |
| Epistylididae | 0 | 0 | 0 | 0 | 0 | 439 | 0 | 0 |  |
| *Rhabdostyla* sp. *** | 0 | 0 | 0 | 0 | 0 | 722 | 0 | 0 |  |
| Vorticellidae | 0 | 0 | 0 | 0 | 0 | 387 | 0 | 0 |  |
| *Trithigmostoma cucullulus ** | 19 | 7 | 0 | 0 | 0 | 141 | 537 | 0 |  |
| *Podophrya fixa ** | 0 | 0 | 0 | 0 | 0 | 0 | 100 | 0 |  |
| Spirotrichea | 0 | 0 | 0 | 0 | 0 | 548 | 1491 | 0 |  |
| Sporadotrichida | 10472 | 280 | 162 | 0 | 55 | 344 | 908 | 0 |  |
| *Halteria* sp. | 0 | 0 | 0 | 0 | 0 | 0 | 4563 | 0 |  |
| Oxytrichidae | 3633 | 6 | 1418 | 0 | 827 | 0 | 0 | 0 |  |
| *Anteholosticha* sp. | 2920 | 0 | 0 | 0 | 0 | 0 | 1021 | 0 |  |
| **PLANTAE** |  |  |  |  |  |  |  |  |  |
| **CHLOROPHYTA** |  |  |  |  |  |  |  |  |  |
| Unknown Chlorophyta | 6993 | 27273 | 674 | 476 | 13630 | 6354 | 96864 | 0 |  |
| **Chlorophyceae** | 299 | 297 | 46 | 163 | 41 | 0 | 159 | 0 |  |
| *Diplosphaera chodatii* | 0 | 0 | 0 | 0 | 0 | 5949 | 0 | 0 |  |
| **Chlamydomonadales** | 4755 | 21433 | 26 | 92 | 1457 | 59 | 1999 | 0 |  |
| *Macrochloris* sp*.* | 0 | 0 | 0 | 0 | 0 | 0 | 6936 | 0 |  |
| *Chlamydomonadaceae* | 1009 | 21199 | 101 | 890 | 4455 | 0 | 1232 | 0 |  |
| *Chlamydomonas* sp. | 56329 | 2423 | 9072 | 4246 | 8325 | 1428 | 25719 | 201 |  |
| *Chloromonas* sp. | 4971 | 1056 | 0 | 233 | 206 | 15 | 76 | 0 |  |
| *Chloromonas hindakii* | 332 | 637 | 0 | 1013 | 414 | 0 | 268 | 0 |  |
| *Chloromonas krienitzii ** | 0 | 0 | 104 | 0 | 0 | 0 | 8 | 0 |  |
| *Chloromonas nivalis* | 2893 | 11379 | 2235 | 18354 | 12568 | 0 | 2480 | 0 |  |
| *Sanguina nivaloides* | 38849 | 113251 | 28220 | 17757 | 8919 | 1377 | 10827 | 97 |  |
| *Chlorococcum oleofaciens** | 0 | 0 | 0 | 0 | 0 | 340 | 0 | 0 |  |
| *Neocystis* sp. | 0 | 0 | 0 | 0 | 0 | 894 | 0 | 0 |  |
| **Scenedesmaceae** | 0 | 0 | 0 | 0 | 0 | 0 | 249 | 0 |  |
| **Trebouxiophyceae** | 15 | 0 | 9 | 0 | 0 | 9410 | 192 | 59 |  |
| **Chlorellales** | 0 | 0 | 0 | 0 | 0 | 0 | 210 | 0 |  |
| *Chlorella* sp. | 0 | 0 | 0 | 0 | 0 | 54 | 362 | 0 |  |
| *Micractinium* sp. | 0 | 0 | 0 | 0 | 0 | 0 | 3882 | 0 |  |
| *Pseudochlorella* sp. | 0 | 0 | 0 | 0 | 0 | 296 | 0 | 0 |  |
| *Pseudochlorella pringsheimii ** | 0 | 0 | 0 | 0 | 0 | 73 | 0 | 0 |  |
| *Coccomyxa* sp. | 0 | 0 | 0 | 0 | 0 | 74 | 0 | 0 |  |
| *Coccomyxa subellipsoidea* | 0 | 0 | 0 | 0 | 0 | 17 | 0 | 0 |  |
| Unknow Microthamniales | 0 | 0 | 0 | 0 | 0 | 1046 | 0 | 0 |  |
| **Prasiolales** | 0 | 0 | 0 | 0 | 0 | 19 | 0 | 0 |  |
| **Koliellaceae** | 220 | 0 | 70 | 0 | 22 | 0 | 115 | 153 |  |
| **Prasiolaceae** | 0 | 0 | 0 | 0 | 0 | 965 | 0 | 0 |  |
| *Prasiola* sp. | 0 | 0 | 0 | 0 | 0 | 9586 | 42850 | 0 |  |
| *Desmococcus olivaceus* | 0 | 0 | 0 | 0 | 0 | 2829 | 0 | 0 |  |
| *Deuterostichococcus epilithicus* | 0 | 0 | 0 | 0 | 0 | 143 | 0 | 0 |  |
| *Stichococcus* sp. | 0 | 0 | 0 | 0 | 0 | 20 | 0 | 0 |  |
| *Stichococcus antarcticus* | 0 | 0 | 0 | 0 | 0 | 24 | 0 | 0 |  |
| *Tritostichococcus coniocybes ** | 0 | 0 | 0 | 0 | 0 | 24 | 0 | 0 |  |
| *Elliptochloris* sp. | 0 | 0 | 0 | 0 | 0 | 113 | 0 | 0 |  |
| **Trebouxiales** |  |  |  |  |  |  |  |  |  |
| **Trebouxiaceae** | 0 | 0 | 0 | 0 | 0 | 73 | 0 | 0 |  |
| *Asterochloris* sp. | 0 | 0 | 0 | 0 | 0 | 86 | 0 | 0 |  |
| *Myrmecia* sp. | 0 | 0 | 0 | 0 | 0 | 4558 | 0 | 0 |  |
| *Trebouxia* sp. | 165 | 0 | 0 | 0 | 96 | 15948 | 0 | 0 |  |
| **Ulotrichales** | 0 | 0 | 0 | 0 | 0 | 1485 | 30 | 0 |  |
| *Kornmannia leptoderma* | 0 | 0 | 0 | 0 | 0 | 191 | 0 | 0 |  |
| *Blidingia* sp. | 0 | 0 | 0 | 0 | 0 | 165 | 0 | 0 |  |
| **STREPTOPHYTA** |  |  |  |  |  |  |  |  |  |
| *Sanionia* sp. | 0 | 0 | 0 | 0 | 0 | 124 | 0 | 0 |  |
| **Pottiaceae** | 0 | 0 | 0 | 0 | 0 | 124 | 0 | 0 |  |
| **PROTOZOA** |  |  |  |  |  |  |  |  |  |
| *Allovahlkampfia* sp. | 0 | 0 | 0 | 0 | 5 | 0 | 195 | 0 |  |
| **EUKARYOTA UNKNOW** | 0 | 0 | 36 | 0 | 0 | 17492 | 2539 | 0 |  |
| Opisthokonta | 0 | 0 | 0 | 0 | 0 | 0 | 38 | 0 |  |
| **UNKNOW** | 0 | 0 | 325 | 0 | 0 | 0 | 42 | 0 |  |
| **TOTAL** | 140281 | 199888 | 43172 | 44177 | 55857 | 84862 | 208779 | 510 |  |
